# Phase Angle (PhA) Is an Easy and Complementary Tool for Assessing Nutritional Status in Ulcerative Colitis (UC) Patients: A Cross-Sectional Study

**DOI:** 10.3390/life14111511

**Published:** 2024-11-20

**Authors:** Viridiana Montsserrat Mendoza-Martínez, Roberto Baños-Vázquez, Guillermo Melendez-Mier, Javier Ivanovychs Carrillo-Rojas, Martha Alison Santoyo-Chávez, Sarahi Ontiveros-López, Annel Gómez-Coello, Galileo Escobedo, Jorge Luis de León-Rendón, Nallely Bueno-Hernández

**Affiliations:** 1 Proteomics and Metabolomics Laboratory, Research Directorate, General Hospital of Mexico “Dr. Eduardo Liceaga”, Mexico City 06720, Mexico; viiriidiios@gmail.com (V.M.M.-M.); robertobanos25@hotmail.com (R.B.-V.); santoyoalison@gmail.com (M.A.S.-C.); gescobedog@msn.com (G.E.); 2 Faculty of Public Health and Nutrition (FASPyN), Autonomous University of Nuevo León, Monterrey 66455, Mexico; melendez651@gmail.com; 3 Specialty Hospital of the National Medical Center “La Raza”, Mexico City 02990, Mexico; ivancarrillorojas@gmail.com (J.I.C.-R.); ontiveros.sarahi@hotmail.com (S.O.-L.); 4 Phoniatrics Department, National Institute of Rehabilitation “Luis Guillermo Ibarra Ibarra”, Mexico City 14389, Mexico; annelgomezc@gmail.com; 5 Inflammatory Bowel Disease Clinic of the Gastroenterology Service of the General Hospital of Mexico “Dr. Eduardo Liceaga”, Mexico City 06720, Mexico; dr.jorgedeleon@hotmail.com

**Keywords:** phase angle, nutritional status, inflammatory bowel disease, ulcerative colitis, bioelectrical impedance

## Abstract

Background: Accumulating evidence has proposed phase angle (PhA) as a marker for assessing cellular integrity and nutritional status in ulcerative colitis (UC) patients; the aim of the study was to evaluate the efficacy of PhA in assessing nutritional status in patients with UC, investigating its potential as a biomarker of disease activity. Methods: We conducted a cross-sectional study in patients with UC and healthy controls. We determined PhA by electrical bioimpedance and categorized participants through bioelectrical impedance analysis. They were classified as normal PhA > 6.1° and low PhA < 6.1° in men and normal PhA > 5.6° and low PhA < 5.6° in women. Results: PhA was significantly lower in UC patients than in controls (5.8 ± 0.8 vs. 6.6 ± 0.7°; *p* < 0.001). Among UC patients, participants with low PhA showed a decrease in lean, dry mass (LDM) (*p* < 0.001), total body water (*p* = 0.008), and intracellular water (*p* = 0.005), accompanied by an increase in extracellular water (*p* = 0.001) compared to UC patients with normal PhA. Conclusions: PhA significantly decreases in UC patients compared to healthy controls and is even more reduced when UC is active. A cut-off point of <6.1 for men and <5.6 for women could be suitable for nutritional diagnosis in patients with UC, but it still needs to be validated.

## 1. Introduction

Inflammatory bowel disease (IBD) is a chronic inflammatory condition, including both Crohn’s disease (CD) and ulcerative colitis (UC). UC is marked by symptoms such as bloody diarrhea stemming from persistent inflammation that begins in the rectum and may spread throughout the colon. In addition to gastrointestinal symptoms, UC often involves extraintestinal manifestations like joint pain, skin and eye complications, anemia, and, notably, malnutrition [1]. Given these complications, nutritional assessment in UC patients is essential. Malnutrition is common due to both the disease’s impact on nutrient absorption and the body’s increased metabolic demands during inflammation. Nutritional evaluation can help identify deficiencies early, allowing for targeted interventions that may improve patient outcomes and quality of life [2].

In UC patients, malnutrition occurs in up to 70% of patients with active disease and up to 38% of patients in remission and is associated with a high number of hospitalizations, frequency of relapses, need for surgery, and postoperative complications [3,4]. These complications are attributed to several factors, including digestive discomfort, pharmacological treatment, abdominal pain, reduced oral intake, inflammatory status, and disease severity. However, there is currently no consensus on the diagnosis of malnutrition in UC patients [5]. The criteria from the Global Leadership Initiative on Malnutrition (GLIM) for the diagnosis of malnutrition in UC are based on the patient’s body mass index (BMI) [6].

However, assessing nutritional status based only on BMI may be inappropriate in UC. In this sense, the European Society for Clinical Nutrition and Metabolism (ESPEN) guidelines, developed to provide evidence-based recommendations for managing clinical nutrition and metabolism in IBD, acknowledge that BMI does not allow the accurate identification of patients with obesity, which often complicates the disease [5], because even patients with increased adiposity may have lower lean mass despite having an average BMI [7].

This information underlines the need to find other markers to provide information about the UC patient’s nutritional status. In fact, there are alternative methods to assess a person’s nutritional status, such as bioelectrical impedance analysis (BIA) and Phase Angle (PhA). In particular, PhA is a bioelectrical impedance parameter that provides insight into cell membrane integrity and cellular health. It reflects the body’s ability to retain electrical charges across cell membranes, which is closely related to cell function and overall nutritional status. In patients with IBD, a lower PhA often indicates compromised cell membrane integrity, potentially due to inflammation, malnutrition, or both [8]. Signori-Urbano et al. found an inverse correlation between the inflammatory markers C-reactive protein (CRP) and erythrocyte sedimentation rate (ESR) and PhA in UC patients [9], suggesting that PhA may help monitor nutritional status in UC patients, serving as a simple, non-invasive tool that can offer valuable information on a patient’s nutritional status and disease progression.

Thus, our primary goal was to evaluate the efficacy of PhA for assessing nutritional status in UC patients while also examining a possible relationship with disease activity.

## 2. Materials and Methods

### 2.1. Trial Design

**Figure 1:**
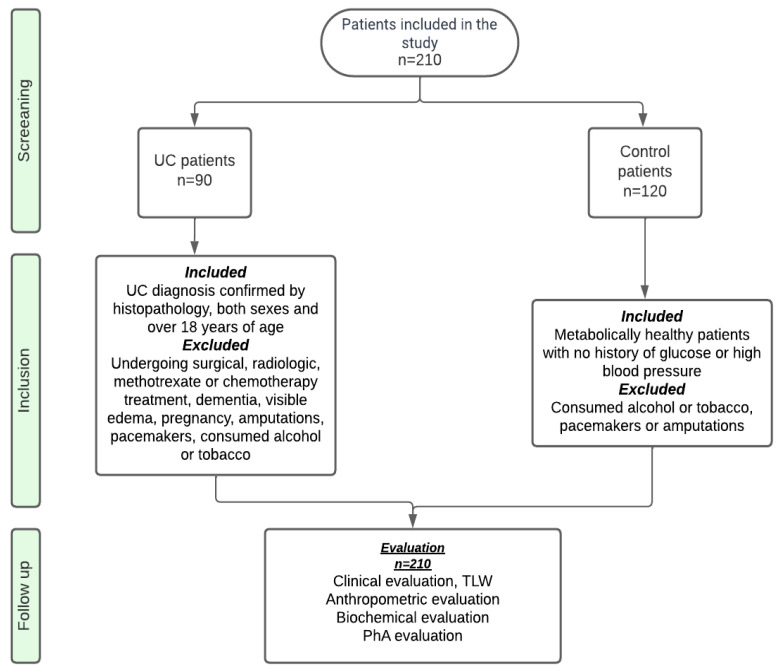
The flowchart illustrates the selection process of the study participants according to the STROBE criteria.

We conducted a multicenter, cross-sectional study according to the STROBE (Observational Studies in Epidemiology) guidelines [10] (Figure 1), enrolling patients between 2020 and 2023 in the General Hospital of Mexico Dr. Eduardo Liceaga (HGMEL) and La Raza Medical Center (RMC). All participants had clinical, anthropometric, and biochemical evaluations.

### 2.2. Ethical Considerations

All participants signed a letter of informed consent and gave their authorization for the publication of the data obtained. The principles of Article 17 of the General Health Law regarding research and the ethical guidelines of the Declaration of Helsinki were followed. The ethical committees of both hospitals approved this project under the numbers DI 19/501/03/098 (HGMEL) and R-2023-3501-079 (RMC), and all participants signed a consent form. We collected data from the protocol registry on ClinicalTrials.gov with the number NCT04143633.

### 2.3. Eligibility Criteria for Participants

We enrolled patients of both sexes above 18 years with an intestinal biopsy-proven diagnosis of UC. We assessed disease activity by True Love and Witts, asking patients about medication use based on their current prescriptions. We excluded from the study participants with surgical, radiologic, methotrexate, or chemotherapy treatment, dementia, visible edema, pregnancy, amputations, and pacemakers. Besides UC patients, we enrolled a healthy control group with no history of high blood glucose, hypertension, or dyslipidemia. We also excluded participants who consumed alcohol or tobacco due to the possible exacerbation of gastrointestinal symptoms. To ensure accurate measurements, participants were required to be fasting, with no prior fluid intake, and were prohibited from intense exercise and alcohol consumption for one day before the assessment.

### 2.4. Outcomes

In the subjects of this research, the aim was to evaluate for the first time the effectiveness of PhA for the assessment of nutritional status by clinical, anthropometric, and biochemical evaluation in UC patients. It also aimed to investigate a possible association with disease activity, whether active or in remission, and to quantify ESR and CRP levels.

### 2.5. Clinical, Anthropometric, and Biochemical Evaluation of Volunteers

We collected and registered all study participants’ demographic, anthropometric, clinical, and laboratory parameters, such as ESR, CRP, cholesterol, triglycerides, lymphocytes, and platelets. We performed BIA analysis and anthropometric assessments by standardized nutritionists using the RJL Quantum IV system (RJL Systems Inc. Clinton Township, MI, USA) to measure body composition and estimate fat mass (FM), lean dry mass (LDM), total body water (TBW), extracellular water (ECW), intracellular water (ICW), and skeletal muscle mass (SMM). The formula for Mexican adults [11] was used to calculate SMM: SMM (kg) = [(Ht2∣*R* × 0.401) + (gender × 3.825) + (age × − 0.071)] + 5.102 [12].

We used Reactance(Xc)/Resistance(R)*(180/π) to estimate PhA [13]. Weight (Weighing Machine, MS 50 SECA; Hamburg, Germany) and height (scale, MD 213 SECA; Hamburg, Germany) were measured, and body mass index (BMI) was calculated using the formula BMI = weight (kg)/height (m)^2. The body circumferences, including waist circumference, were measured by placing a tape (Lufkin tape measure 6 mm × 2 m diameter pocket tape) between the iliac crest and the lumbar rib. We measured hip circumference by placing the tape at the predominant point of the buttocks, following the National Institute of Public Health recommendations [14]. To ensure accurate measurements, participants were required to be fasting, with no prior fluid intake, and were prohibited from intense exercise and alcohol consumption one day before the assessment. Biochemical parameters were extracted from the patient’s electronic medical records, including results from standard blood tests relevant to the study.

### 2.6. Sample Size

We estimated the sample size using the GPower v.3.1 9.2 program, assuming an effect size of 0.4, an alpha error of 0.05, and a power of 80%. This resulted in a sample size of 90 participants per group. We included an additional 10% of patients to account for possible losses. We decided to increase the number of participants to 120 in the control group to improve the study’s statistical power and detect genuine associations in both groups.

### 2.7. Statistical Analysis

We categorized UC patients according to the PhA value. For women, a PhA < 5.5° was classified as a “low PhA patient group with PhA < 6.1°” and a PhA > 5.6° as a “normal PhA patient group”. For men, the cutoff was maintained at 6.1°, consistent with PhA > 6.1°, following Peng et al.’s 2022 guidelines. We used the PhA cutoff value proposed by the GLIM criteria as the gold standard [15]. After assessing the normality of data by the Kolmogorov–Smirnov test, we performed the Mann–Whitney U and *t*-test to compare data between UC patients and healthy controls. We performed bivariate associations by the Pearson correlation coefficient (r) for body composition variables with PhA in UC patients, defining null correlation values as ranging from 0.00 to 0.09, weak correlations from 0.10 to 0.29, moderate correlations from 0.30 to 0.49, and strong correlations from 0.50 to 1.0, according to the guidelines of Hernández Lalinde J.D. et al. [16]. We used the statistical package IBM-SPSS version 23 (IBM, Armonk, NY, USA) for the analysis, considering a *p*-value < 0.05 as significant.

## 3. Results

Two hundred and ten patients met the inclusion criteria and agreed to participate in the study. There were ninety UC patients and one hundred and twenty healthy controls; the average age of participants in both groups was 30 ± 12 years, and they were predominantly women (62%) (Table 1).

### 3.1. Differences Between UC Patients and Controls

Concerning disease activity, we found that up to 35% of UC patients were in remission, and 65% had active disease. While 100% of UC patients took mesalazine, almost 8% of them also took steroids. UC patients had higher platelet count (307.1 ± 144.2 vs. 249.4 ± 48.8 K/µL, *p* < 0.001) and lower resistance (629.1 ± 99.1 vs. 692.7 ± 110.7, *p* < 0.001), reactance (64.4 ± 9.9 vs. 80 ± 10.7, *p* < 0.001), weight (61.6 ± 11.9 vs. 65.4 ± 12.8 kg, *p* = 0.043), LDM (11.1 ± 3.1 vs. 17.2 ± 4.3 kg, *p* < 0.001), and SMM (18.2 ± 5.3 vs. 21.4 ± 4.9 kg, *p* < 0.001) compared to controls. In addition, PhA was significantly lower in UC patients than in controls (5.9 ± 0.9 vs. 6.6 ± 0.7°, *p* < 0.001). We found no differences in BMI between UC patients and controls (24.2 ± 3.9 vs. 24.1 ± 3.4 kg/m^2^, *p* = 0.534) (Table 1). Of the patients with UC, 52.2% had a low PhA. Among these, 49% were women and 51% were men. In contrast, in the control group, eight patients had a low PhA, with five being women and three being men.

### 3.2. Phase Angle and Disease Activity

When comparing patients with active disease to those in remission, we observed notable differences in CRP levels (11.7 ± 18.3 vs. 4.1 ± 3.7 mg/L, *p* = 0.044) (Table 2). However, we found no significant differences in other biochemical parameters. Furthermore, we found no differences in BMI (24.4 ± 4.2 vs. 24.2 ± 3.4 kg/m^2^, *p* = 0.696) or PhA (5.9 ± 1 vs. 5.9 ± 0.9°, *p* = 0.853) when comparing UC patients with active disease to UC patients in remission. Conversely, there were significant differences in PhA when comparing UC patients with healthy controls (5.9 ± 0.9 vs. 6.6 ± 0.7°, *p* < 0.001) (Table 1).

Comparing patients with low PhA and those with normal PhA values, we found a decrease in LDM (10.1 ± 2.7 vs. 13.1 ± 2.4 kg, *p* < 0.001 in patients with active disease; 8.9 ± 2.2 vs. 12.1 ± 3.6 kg, *p* = 0.008 in patients in remission), SMM (14.9 ± 3.6 vs. 21.7 ± 5.8 kg, *p* < 0.001 in patients with active disease; 15.7 ± 3.3 vs. 20.7 ± 3.5 kg, *p* < 0.001 in patients in remission), and ICW (13.1 ± 3.3 vs. 17.6 ± 5.2 L, *p* < 0.001 in patients with active disease; 15.1 ± 4.1 vs. 17.3 ± 3.3 L, *p* = 0.103 in patients in remission). Only patients with active disease and low PhA exhibited a decrease in weight (57.7 ± 13.4 vs. 65.2 ± 12 kg, *p* = 0.030), TBW (25.7 ± 5.6 vs. 31.8 ± 8.2 L, *p* = 0.002), and ECW (12.3 ± 2.6 vs. 14.1 ± 3.1 L, *p* < 0.001) compared to patients with normal PhA. Conversely, patients with active disease and low PhA showed a significant increase in ESR (22.7 ± 14.4 vs. 15.1 ± 13.7 mm/h, *p* = 0.050) and CRP (17.8 ± 23.9 vs. 5.6 ± 6.2 mg/L, *p* = 0.013) compared to patients with normal PhA (Table 3).

In the study, the distribution of PhA among UC patients and controls revealed distinct patterns. Among UC patients with active disease, 52.5% had a low PhA, while 47.5% had a normal PhA. In contrast, among UC patients in remission, 51.6% had a low PhA, and 48.4% had a normal PhA (Table 3).

### 3.3. Correlations of Phase Angle with Body Composition Markers

**Figure 2:**
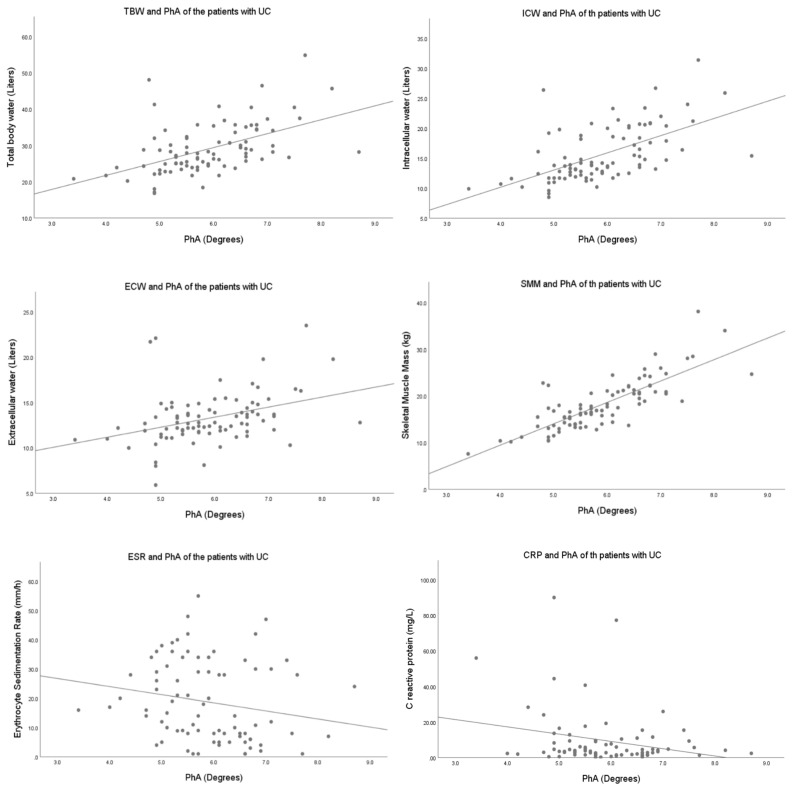
Correlations between PhA, body composition and biochemical parameters in UC patients. TBW: total body water, ICW: intracellular water, ECW: extracellular water, CRP: C-reactive protein, ESR: erythrocyte sedimentation rate, PhA: phase angle, UC: ulcerative colitis.

We found a significant correlation between TBW (r = 0.509; *p* < 0.001), ICW (*r* = 0.585; *p* < 0.001), ECW (r = 0.366; *p* < 0.001), SMM (r = 0.803; *p* < 0.001), and LDM (r = 0.656; *p* < 0.001) with PhA in UC patients. Similar results were observed in the control group: TBW (r = 0.493; *p* < 0.001), ICW (r = 0.558; *p* = 0.001), ECW (r = 0.342; *p* < 0.001), SMM (r = 0.704; *p* < 0.001), and LDM (r = 0.617; *p* < 0.001) (Figure 2).

## 4. Discussion

**Figure 3:**
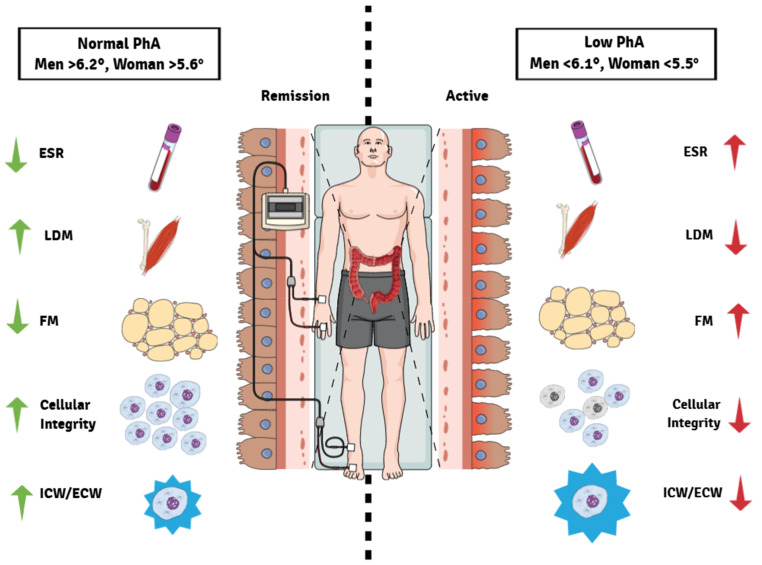
Proposal of the main metabolic changes in UC patients with low PhA. The metabolic alterations in UC patients with low PhA are associated with disease activity, increased ESR, decreased lean, dry mass, increased fat, and increased fluid retention. ESR: erythrocyte sedimentation rate, PhA: phase angle, TBW: total body water, ICW: intracellular water, ECW: extracellular water, UC: ulcerative colitis. Red arrows indicate that changes are detrimental. Green arrows indicate that changes are beneficial.

PhA is considered a marker of cellular integrity and is directly related to prognosis and mortality in numerous diseases [16]. Furthermore, it serves as a predictor of malnutrition, which is defined by The World Health Organization (WHO) as a deficiency, excess, or imbalance in a person’s intake of energy and/or nutrients [17]. However, there are few studies examining PhA in patients with IBD, primarily focused on CD. Peng et al. studied 169 CD patients, finding that PhA = 6.01° in men and 5.2° in women is associated with malnutrition. We found similar PhA values in UC patients, which suggests that low PhA may be a marker of the disease (Figure 3) [15].

The findings in CD are possible because the disease is transmural, leading to increased cell death and decreased nutrient absorption, thus magnifying malnutrition compared to that observed in UC patients. However, this phenomenon could also occur in UC patients due to medication use, disease activity, and poor nutrient absorption. The same research team mentioned that patients with PhA < 6.1° in men and <5.5 in women have a higher risk of malnutrition as a manifestation of the inflammatory process (Figure 3). So far, this finding aligns with the study by Coffi et al. (2020), which also reported that lower PhA values were indicative of poorer nutritional status and higher levels of inflammation in IBD patients [18]. Otherwise, these data are consistent with those reported by Więch et al., where they showed that IBD children and adolescents with low PhA (<5°) display poor nutritional and functional status compared to healthy subjects [19]. In addition, low PhA is associated with reduced exercise capacity and physical activity in IBD patients. Normando C. et al. showed that low PhA correlates with decreased daily activities, especially in the elderly (Figure 3) [20]. In UC, the behavior is similar, as patients reduce their physical activity for fear of losing weight and muscle mass, which could also explain the decrease in PhA in our study population.

In addition, Cioffi et al. pointed out that inflammation and malnutrition negatively affect the electrical properties of tissue, leading to decreased PhA in UC patients compared to healthy individuals [21,22]. This phenomenon occurs because acute inflammation is the body’s response to damage under transitory conditions; however, when inflammation becomes chronic, as in IBD patients, tissue damage and physiological imbalance occur due to the abnormal production of proinflammatory cytokines [22]. Inflammation related to disease activity leads to low PhA. In our population of UC patients, lower PhA was more evident in patients with active disease than in patients in remission, and this reduction was more significant in women. Thus, active disease and female sex can be considered factors related to malnourishment in UC; nevertheless, we need to conduct further studies before making solid conclusions [15].

Urbano et al. showed that PhA negatively correlates with CRP and ESR in UC patients, a finding also observed herein. Chronic inflammation contributes to metabolic abnormalities, changes in body composition, and chronic diseases such as sarcopenia, diabetes, and metabolic syndrome [9,21]. In our study, UC patients with low PhA (<6.1°) had lower weight, LDM, water, and ICW, accompanied by higher FM, compared to patients with normal PhA and regardless of disease activity [23]. However, inflammation and disease activity can affect fluid levels and influence PhA. According to the study by Bongiolo et al., PhA may reflect muscle mass loss independently of the disease activity. In our study, patients with UC who had low PhA (<6.1°) had lower weight, lower LDM, less water, especially ICW, and a higher amount of FM regardless of disease activity compared to patients with regular PhA (Figure 3) [23]. Based on the study of Bongiolo et al., PhA can reflect muscle mass depletion independently of active disease [24]. Many known risk factors for IBD trigger the loss of muscle and fat reserves, altering cellular fluid distribution. This change in body composition is associated with the prognosis, severity, and stage of UC [25]. We want to note that none of our patients had low BMI, which strengthens the argument for using other parameters, such as PhA, to monitor nutritional status in UC. The applicability of these data is helpful for physicians and nutritionists, as they can make a more objective assessment based on the activity of the disease and not only on the BMI. Interestingly, 40% of patients in our study population were overweight. However, the analysis of body composition and PhA revealed that patients could be at risk of malnutrition or sarcopenia [20]. In our study, patients with low PhA and active disease had higher CHF, an index related to cardiovascular risk. Although CHF levels do not determine risk, chronic inflammation leads to several cardiovascular and metabolic events, as well as sarcopenia, malnutrition, and poor nutrient absorption, especially in women compared to men [25,26,27,28].

In this study, we found changes related to the amount of ICW and ECW in UC patients compared to controls. This phenomenon may occur because low PhA is associated with altered distribution of ECW, hemoglobin, hematocrit, and muscle mass [26], as well as an imbalance of TBW and ECW in patients with cancer [29].

Several studies have shown that patients with IBD have lower levels of LDM, an indicator of the amount of minerals, visceral proteins, and muscles, particularly in pediatric patients [30]. Herein, we observed that UC patients display a significant decrease in LDM compared to controls. Since LDM also decreased in UC patients with active disease, we speculate that the amount of muscle and visceral proteins may decrease as disease severity and inflammation increase. A study conducted by Dhillon RJ et al. reported a decrease in muscle mass associated with the release of inflammatory mediators, such as tumor necrosis factor-alpha (TNF-α) and interleukin-6 (IL-6) [22,24,31]. Moreover, Bellido et al. emphasized that standardizing test protocols will facilitate the diagnosis and assessment of the risk associated with reduced PhA and aid in evaluating responses to therapeutic interventions [32]. Our results align with previous reports, showing that inflammatory markers such as ESR and CRP increase according to disease activity and PhA [33].

Regardless of disease activity, PhA may represent an alternative method to assess nutritional status in UC patients during active disease or remission to avoid malnourishment. Moreover, considering PhA may help improve the subjective results using non-specific measurements such as BMI or waist circumference. These traditional measures cannot accurately identify individuals with overt malnutrition who require short-term dietary support. Data from this study are relevant and may contribute to implementing personalized nutritional therapy that could lead to a better disease prognosis in UC. In parallel, a long-term follow-up of the patients could improve the study, which may also allow us to increase the sample size to recalculate parameters that did not reach significance but showed a trend. Additionally, we did not measure the amount of skeletal muscle and only registered LDM, a measurement that can be affected by the patient’s bone mineral density. This study may open new research avenues focused on detecting and preventing malnutrition in UC patients. Future longitudinal studies on PhA in UC patients should include a diverse cohort and standardized measurement protocols to ensure consistency. Increasing the sample size will help minimize random variation. Regular, long-term follow-up is needed to correlate PhA changes with clinical outcomes like disease activity and quality of life. Appropriate statistical methods should be used to analyze trends and control for confounding variables. These steps will enhance the understanding of PhA’s prognostic value in UC management.

## 5. Conclusions

We found that PhA significantly decreases in UC patients, particularly those with active disease. These findings suggest that PhA may help detect inflammatory activity and monitor changes in body composition in UC patients.

PhA appears to work as a complementary tool to assess nutritional status in UC patients when considering PhA < 6.1° to identify individuals with high inflammatory response and low LDM levels.

PhA exhibits correlations with inflammatory parameters that persist even after disease remission, which aligns with previous reports. It is worth noting that our results did not find significant differences in patients’ BMI, confirming that this parameter is not strong enough to detect UC-related malnutrition. This finding reveals how relevant it may be to incorporate PhA in assessing nutritional status in UC patients. We still need to conduct prospective studies to evaluate the cutoff for different disease stages and validate it in this population.

## Figures and Tables

**Table 1:** Demographic, biochemical, and body composition parameters in the study population.

	Total Population (n = 210)	UC Patients (n = 90)	Control (n = 120)	*p*-Value
Age, years (mean ± SD)	30 ± 12	39 ± 12	23 ± 3	<0.001 *
Gender, n (%)				
Men	90 (38)	43 (36)	47 (39)	0.134
Women	120 (62)	47 (64)	73 (61)	
Weight, kg (mean ± SD)	64 ± 12.4	62 ± 11.9	65.4 ± 12.8	0.043 *
Waist, cm (mean ± SD)	81.1 ± 9.9	82.5 ± 10.4	80.3 ± 9.5	0.085
Hip, cm (mean ± SD)	97.9 ± 8.1	97.4 ± 7.1	98.2 ± 7.4	0.596
BMI, kg/m^2^ (mean ± SD)	24.2 ± 3.6	24.2 ± 3.9	24.1 ± 3.4	0.534
Cholesterol, mg/dL (mean ± SD)	168.1 ± 37.1	172.1 ± 45.1	165.4 ± 29.5	0.224
Triglycerides, mg/dL (mean ± SD)	110.8 ± 75.9	118.9 ± 75.6	104.8 ± 75.1	0.186
Lymphocytes, count (mean ± SD)	2038.1 ± 662.7	2150.8 ± 856.8	1963.5 ± 465.6	0.065
Platelets K/µL (mean ± SD)	273.5 ± 103.8	307.1 ± 144.2	249.4 ± 48.8	<0.001 *
Resistance, ohms (mean ± SD)	665.5 ± 110.2	629.1 ± 99.1	692.7 ± 110.7	<0.001 *
Reactance, ohms (mean ± SD)	73.3 ± 12.9	64.4 ± 9.9	80 ± 10.7	<0.001 *
Fat, kg (mean ± SD)	20.3 ± 7.3	21.8 ± 8.1	23.6 ± 6.8	0.326
LDM, kg (mean ± SD)	14.1 ± 3.5	11.1 ± 3.1	17.2 ± 4.3	<0.001 *
SMM, kg (mean ± SD)	20.1 ± 5.3	18.2 ± 5.3	21.4 ± 4.9	<0.001 *
TBW L (mean ± SD)	28.4 ± 6.8	29.1 ± 7.1	30.1 ± 6.7	0.412
ICW, L (mean ± SD)	15.5 ± 4.4	15.6 ± 4.5	57.1 ± 3.1	0.729
ECW, L (mean ± SD)	13 ± 2.7	13.2 ± 2.8	42.9 ± 3.1	0.235
PhA, degrees (mean ± SD)	6.3 ± 0.9	5.9 ± 0.9	6.6 ± 0.7	<0.001 *

BMI: Body Mass Index, UC: Ulcerative Colitis. SD: Standard Deviation, TBW: Total Body Water, ICW: Intracellular Water, ECW: Extracellular Water, L: liters, mg: milligrams, dL: deciliter, PhA: Phase Angle, K/µL: thousand per microliter, LDM: lean dry mass SD: Standard Deviation, *t*-test for independent samples. * Significant *p*-value < 0.05.

**Table 2:** Metabolic parameters in UC patients according to disease activity.

	Active Disease (n = 58)	Remission (n = 32)	*p*-Value
Weight, kg (mean ± SD)	61.4 ± 13.4	62.7 ± 9.2	0.308
Waist, cm (mean ± SD)	97.8 ± 8.5	82.5 ± 11.5	0.671
Hip, cm (mean ± SD)	97.3 ± 9.7	97.8 ± 8.5	0.664
BMI, kg/m^2^ (mean ± SD)	24.5 ± 4.2	24.1 ± 3.3	0.593
PhA, degrees (mean ± SD)	5.8 ± 0.9	5.9 ± 0.9	0.646
FMI, kg/m^2^ (mean ± SD)	9.1 ± 4.1	8.7 ± 3.4	0.768
FMSMM kg/m^2^ (mean ± SD)	7.1 ± 1.5	6.9 ± 1.2	0.636
ESR, mm/h (mean ± SD)	19.1 ± 14.5	18.1 ± 12.8	0.989
CRP, mg/L (mean ± SD)	11.7 ± 18.3	4.1 ± 3.7	0.044 *
Cholesterol, mg/dL (mean ± SD)	171.3 ± 42.4	173.5 ± 50.6	0.652
Triglycerides, mg/dL (mean ± SD)	124.9 ± 86.3	107.7 ± 48.8	0.394
Lymphocytes, count(mean ± SD)	2163.7 ± 871.7	2126.5 ± 842.5	0.933
Platelets K/µL (mean ± SD)	320.6 ± 167.5	281.2 ± 80.5	0.635
Fat, kg (mean ± SD)	21.6 ± 8.3	22.7 ± 7.8	0.409
LDM, kg (mean ± SD)	11.6 ± 3	10.3 ± 3.4	0.072
SMM, kg (mean ± SD)	18.3 ± 5.9	18.1 ± 4.3	0.799
TBW L (mean ± SD)	28.8 ± 7.6	29.6 ± 5.9	0.268
ICW, L (mean ± SD)	13.2 ± 3.1	13.4 ± 2.5	0.188
ECW, L (mean ± SD)	13.2 ± 3.1	13.4 ± 2.5	0.254

BMI: Body Mass Index, UC: Ulcerative Colitis, ESR: Erythrocyte Sedimentation Rate, PhA: Phase Angle, CRP: C-Reactive Protein, TBW: Total Body Water, ICW: Intracellular Water, ECW: Extracellular Water, L: liters, mg: milligrams, dL: deciliter, cm: centimeters, SD: Standard Deviation, * Significant *p*-value < 0.05. The statistical analyzes were done with *t*-test & Mann–Whitney U test.

**Table 3:** Differences between UC patients experiencing active disease or remission are categorized by low and normal PhA.

	Active Disease			Remission		
	Low PhA	Normal PhA	*p*-Value	Low PhA	Normal PhA	*p*-Value
	(n = 37)	(n = 21)		(n = 19)	(n = 13)	
Age, years (mean ± SD)	37 ± 11	37 ± 9	0.876	49 ± 13	40 ± 15	0.134
Weight, kg (mean ± SD)	57.9 ± 13.1	68.5 ± 11.2	0.004 *	61.8 ± 10.2	64.1 ± 7.8	0.529
Waist, cm (mean ± SD)	81.4 ± 11.6	84.7 ± 10.7	0.304	82.9 ± 9.1	82.1 ± 0.5	0.816
Hip, cm (mean ± SD)	95.9 ± 10.7	100.1 ± 7.1	0.127	98.1 ± 9.8	97.4 ± 6.8	0.814
WC: Waist-to-Hip Ratio (mean ± SD)						
Women	0.86 ± 0.06	0.87 ± 0.08	0.805	0.83 ± 0.06	0.84 ± 0.12	0.906
Men	0.82 ± 0.09	0.81 ± 0.08	0.863	0.86 ± 0.09	0.84 ± 0.06	0.680
Total Cholesterol, mg/dL (mean ± SD)	167.3 ± 42.2	179.7 ± 43.7	0.310	177.1 ± 54.4	168.5 ± 46.5	0.658
Triglycerides, mg/dL (mean ± SD)	114.7 ± 47.3	146.1 ± 131.6	0.208	105.1 ± 51.3	111.6 ± 47.1	0.729
Lymphocytes count (mean ± SD)	2206.1 ± 876.8	2115.4 ± 896.7	0.721	2129.9 ± 999.4	2121.7 ± 595	0.980
Platelets K/µL (mean ± SD)	326.9 ± 167.8	318.3 ± 170.9	0.859	276.1 ± 93.5	288.8 ± 60.6	0.688
ESR, mm/h (mean ± SD)	23.1 ± 13.4	12.4 ± 14.1	0.008 **	17.9 ± 13.4	18.3 ± 12.4	0.939
CRP, mg/L (mean ± SD)	15.6 ± 21.8	5.4 ± 6.2	0.015 *	3.1 ± 2.1	5.5 ± 5.2	0.119
Resistance, ohms (mean ± SD)	681.4 ± 100.3	587.5 ± 91.5	0.001	643.1 ± 97.8	590.7 ± 59.3	0.084
Reactance, ohms (mean ± SD)	61.7 ± 9.3	67.6 ± 9	0.001	58.9 ± 8.2	69.6 ± 10.6	0.004 *
Fat, kg (mean ± SD)	22.3 ± 9.1	20.2 ± 6.7	0.356	24.7 ± 7.5	19.9 ± 7.6	0.104
LDM, kg (mean ± SD)	10.2 ± 2.5	14.1 ± 2.1	<0.001 **	8.7 ± 2.3	12.5 ± 3.6	0.002 **
SMM, kg (mean ± SD)	15.3 ± 3.4	23.9 ± 5.4	<0.001 **	15.6 ± 3.1	28.3 ± 6.6	<0.001 **
TBW L (mean ± SD)	25.8 ± 5.5	34.2 ± 8.1	<0.001 **	28.3 ± 6.6	31.4 ± 4.3	0.166
ICW, L (mean ± SD)	13.2 ± 3.3	19.2 ± 5.1	<0.001 **	14.9 ± 3.9	17.9 ± 3.1	0.041 **
ECW, L (mean ± SD)	12.3 ± 2.5	14.9 ± 3.1	0.001 **	13.3 ± 2.9	13.5 ± 1.8	0.839

ICC: Waist-to-Hip Ratio, BMI: Body Mass Index, UC: Ulcerative Colitis, ESR: Erythrocyte Sedimentation Rate, CRP: C-reactive protein, LDM: Lean Dry Mass, TBW: Total Body Water, ICW: Intracellular Water, ECW: Extracellular Water, SMM: Skeletal Muscles Mass, L: liters, mg: milligrams, dL: deciliter, cm: centimeters, PhA: Phase Angle. PhA low: <6.1°, PhA normal: >6.1° SD: Standard Deviation, * Significant *p*-value < 0.05 with independent samples *t*-test & ** Significant *p*-value < 0.05 with Mann–Whitney U test.

## Data Availability

The data supporting the findings of this study are available on the link: “C:\Users\nalle\Dropbox\HospitalGeneral\1PROTOCOLOS\AF-CUCI\Basede datos\Base de datos CUCI y controles.sav”.

## References

[1] Zhang Y.Z., Li Y.Y. (2014). Inflammatory bowel disease: Pathogenesis. World J. Gastroenterol..

[2] Gompertz M., Sedano R. (2019). Manifestaciones clínicas y endoscópicas en enfermedad inflamatoria intestinal. Rev. Médica Clínica Las Condes.

[3] Li S., Ney M., Eslamparast T., Vandermeer B., Ismond K.P., Kroeker K., Halloran B., Raman M., Tandon P. (2019). Systematic review of nutrition screening and assessment in inflammatory bowel disease. World J. Gastroenterol..

[4] Nishikawa H., Nakamura S., Miyazaki T., Kakimoto K., Fukunishi S., Asai A., Nishiguchi S., Higuchi K. (2021). Inflammatory Bowel Disease and Sarcopenia: Its Mechanism and Clinical Importance. J. Clin. Med..

[5] Bischoff S.C., Bager P., Escher J., Forbes A., Hébuterne X., Hvas C.L., Joly F., Klek S., Krznaric Z., Ockenga J. (2023). ESPEN guideline on Clinical Nutrition in inflammatory bowel disease. Clin. Nutr..

[6] Fiorindi C., Luceri C., Dragoni G., Piemonte G., Scaringi S., Staderini F., Nannoni A., Ficari F., Giudici F. (2020). GLIM Criteria for Malnutrition in Surgical IBD Patients: A Pilot Study. Nutrients.

[7] Forbes A., Escher J., Hébuterne X., Kłęk S., Krznaric Z., Schneider S., Shamir R., Stardelova K., Wierdsma N., Wiskin A.E. (2017). ESPEN guideline: Clinical nutrition in inflammatory bowel disease. Clin. Nutr..

[8] Llames L., Baldomero V., Iglesias M.L., Rodota L.P. (2013). Values of the phase angle by bioelectrical impedance; nutritional status and prognostic value. Nutr. Hosp..

[9] Urbano A.P.S., Sassaki L.Y., Dorna M.S., Presti P.T., Carvalhaes M., Martini L.A., Ferreira A.L.A. (2018). Associations among body composition, inflammatory profile and disease extent in ulcerative colitis patients. Rev. Assoc. Med. Bras. (1992).

[10] Vandenbroucke J.P., Von Elm E., Altman D.G., Gotzsche P.C., Mulrow C.D., Pocock S.J., Poole C. (2009). Strengthening the reporting of observational studies in epidemiology (STROBE): Explanation and elaboration. Gac. Sanit..

[11] Macias N., Aleman-Mateo H., Esparza-Romero J., Valencia M.E. (2007). Body fat measurement by bioelectrical impedance and air displacement plethysmography: A cross-validation study to design bioelectrical impedance equations in Mexican adults. Nutr. J..

[12] Janssen I., Heymsfield S.B., Baumgartner R.N., Ross R. (2000). Estimation of skeletal muscle mass by bioelectrical impedance analysis. J. Appl. Physiol. (1985).

[13] Siddiqui N.I., Khan S.A., Shoeb M., Bose S. (2016). Anthropometric Predictors of Bio-Impedance Analysis (BIA) Phase Angle in Healthy Adults. J. Clin. Diagn. Res..

[14] Rojas M.C., Vargas R.V. (2004). La medición de la talla y el peso. guía para el personal de la salud del primer nivel de atención.

[15] Peng Z., Xu D., Li Y., Peng Y., Liu X. (2022). Phase Angle as a Comprehensive Tool for Nutritional Monitoring and Management in Patients with Crohn’s Disease. Nutrients.

[16] Lalinde J.D.H., Castro F.E., Rodríguez J.E., Rangel J.G.C., Sierra C.A.T., Torrado M.K.A., Bermúdez Pirela V.J. (2018). Sobre el uso adecuado del coeficiente de correlación de Pearson: Definición, propiedades y suposiciones. Arch. Venez. De Farmacol. Ter..

[17] Sellen D. (1995). Physical status: The use and interpretation of anthropometry. Report of a WHO Expert Committee. World Health Organ Tech. Rep. Ser..

[18] Cioffi I., Marra M., Imperatore N., Pagano M.C., Santarpia L., Alfonsi L., Testa A., Sammarco R., Contaldo F., Castiglione F. (2020). Assessment of bioelectrical phase angle as a predictor of nutritional status in patients with Crohn’s disease: A cross sectional study. Clin. Nutr..

[19] Wiech P., Dabrowski M., Bazalinski D., Salacinska I., Korczowski B., Binkowska-Bury M. (2018). Bioelectrical Impedance Phase Angle as an Indicator of Malnutrition in Hospitalized Children with Diagnosed Inflammatory Bowel Diseases—A Case Control Study. Nutrients.

[20] Norman K., Stobäus N., Fau-Pirlich M., Pirlich M., Fau-Bosy-Westphal A., Bosy-Westphal A. (2012). Bioelectrical phase angle and impedance vector analysis—Clinical relevance and applicability of impedance parameters. Clin. Nutr..

[21] Mentella M.C., Scaldaferri F., Pizzoferrato M., Gasbarrini A., Miggiano G.A.D. (2019). The Association of Disease Activity, BMI and Phase Angle with Vitamin D Deficiency in Patients with IBD. Nutrients.

[22] da Silva B.R., Orsso C.E., Gonzalez M.C., Sicchieri J.M.F., Mialich M.S., Jordao A.A., Prado C.M. (2023). Phase angle and cellular health: Inflammation and oxidative damage. Rev. Endocr. Metab. Disord..

[23] Jablonska B., Mrowiec S. (2023). Nutritional Status and Its Detection in Patients with Inflammatory Bowel Diseases. Nutrients.

[24] Bongiolo A.M., Machado M.J., Lazarotto B.A., Rupp M.L.C., Dal-Pizzol F., Pires M.M.S. (2024). Phase Angle as a Predictor of Muscle Mass in Patients with Inflammatory Bowel Disease. Arq. Gastroenterol..

[25] Casirati A., Crotti S., Raffaele A., Caccialanza R., Cereda E. (2023). The use of phase angle in patients with digestive and liver diseases. Rev. Endocr. Metab. Disord..

[26] Ryan E., McNicholas D., Creavin B., Kelly M.E., Walsh T., Beddy D. (2019). Sarcopenia and Inflammatory Bowel Disease: A Systematic Review. Inflamm. Bowel Dis..

[27] Nunez P., Garcia Mateo S., Quera R., Gomollon F. (2021). Inflammatory bowel disease and the risk of cardiovascular diseases. Gastroenterol. Hepatol..

[28] Cao Q., Yu S., Xiong W., Li Y., Li H., Li J., Li F. (2018). Waist-hip ratio as a predictor of myocardial infarction risk: A systematic review and meta-analysis. Medicine.

[29] Katsura N., Yamashita M., Ishihara T. (2021). Extracellular water to total body water ratio may mediate the association between phase angle and mortality in patients with cancer cachexia: A single-center, retrospective study. Clin. Nutr. ESPEN.

[30] Thangarajah D., Hyde M.J., Konteti V.K., Santhakumaran S., Frost G., Fell J.M. (2015). Systematic review: Body composition in children with inflammatory bowel disease. Aliment. Pharmacol. Ther..

[31] Dhillon R.J., Hasni S. (2017). Pathogenesis and Management of Sarcopenia. Clin. Geriatr. Med..

[32] Bellido D., García-García C., Talluri A., Lukaski H.C., García-Almeida J.M. (2023). Future lines of research on phase angle: Strengths and limitations. Rev. Endocr. Metab. Disord..

[33] Sands B.E. (2015). Biomarkers of Inflammation in Inflammatory Bowel Disease. Gastroenterology.

